# Platelet Apoptosis in Patients with Coronary Artery Disease Before and After CABG

**DOI:** 10.3390/ijms27073304

**Published:** 2026-04-06

**Authors:** Arkadiy A. Metelkin, Ekaterina A. Sergeeva, Mikhail A. Popov, Dmitriy I. Zybin, Dmitriy V. Shumakov, Sergey G. Morozov, Alisa A. Sokolovskaya

**Affiliations:** 1 Department of Molecular and Cellular Pathophysiology, Research Institute of General Pathology and Pathophysiology, Baltiyskaya 8, 125315 Moscow, Russia; 2 M.F. Vladimirsky Moscow Regional Clinical Research Institute, Schepkina St. 61/2, 129110 Moscow, Russia

**Keywords:** apoptosis, platelets, cardiovascular disease, coronary heart disease

## Abstract

Cardiovascular diseases have been one of the leading causes of death worldwide for over 30 years and coronary artery disease occupies a prominent place among them. Predicting the long-term outcome after coronary artery bypass graft (CABG) is still a challenging task; however, since platelets are directly involved in the course of the disease, their functional status can help predict postoperative complications. The objective was to evaluate the dynamics of platelet apoptosis before and after CABG. The study involved 30 patients with a scheduled CABG suffering from coronary artery disease. Flow cytometry, Western blot and polymerase chain reaction were used to evaluate apoptosis and the activation of platelets. Changes in platelet membranes and the depolarization of mitochondrial membranes were observed, as well as the expression of active caspase 8 and BCL-2, indicating the activation of apoptosis through an extrinsic receptor-dependent signaling pathway. The data obtained suggest significant changes in platelet metabolic processes, which could play a crucial role in the development of coronary artery disease and atherosclerosis as well as being associated with postoperative complications.

## 1. Introduction

Cardiovascular diseases (CVDs) have been one of the leading causes of death worldwide for over 30 years. The mortality rate from CVD has increased from 13.1 million in 1990 to 19.2 million in 2023, reaching 20.5 million people, one third of all deaths worldwide [1]. Among the diseases of the circulatory system, coronary artery disease (CAD) occupies a prominent place. Complications of CAD, such as myocardial infarction, account for 44% of CVD deaths [2].

The main surgical methods for treating coronary artery disease are percutaneous coronary intervention and coronary artery bypass graft [3]. Predicting the long-term outcomes and complications after surgery, such as restenosis, is one of the biggest challenges in interventional cardiology [4]. Researchers are currently searching for effective markers that can predict the clinical outcome of patients undergoing myocardial revascularization surgery [5,6,7,8]. One potential marker is the functional state of platelets, which are small blood cells that play an important role in the formation of blood clots during vascular events and in inflammation associated with thrombosis, interstitial edema, embolization of the microcirculatory bed and cell death [9,10,11,12]. The functional state of platelets can be assessed using the phenomenon of apoptosis, or programmed cell death.

Previously, it was thought that apoptosis only occurred in nuclear cells. However, recent studies have shown that platelets and red blood cells also undergo apoptosis under the influence of certain environmental factors, as well as during the completion of their physiological life span, representing a form of programmed nuclear cell death [13,14,15]. A number of intracellular events are involved in apoptosis, including the externalization of phosphatidylserine to the outside of the platelet membrane, a decrease in mitochondrial potential, the activation of caspases 3, 8, and 9, and the activation or inhibition of proteins involved in the process. These include proapoptotic proteins such as BAX, BAK, and cytochrome c, and antiapoptotic proteins like BCL-2 and BCL-XL [15,16].

Recently, there has been an increasing interest in the mechanisms of platelet activation and apoptosis in various diseases [17,18,19], including those of the cardiovascular system [20,21]. Dysregulation of these processes has been implicated in a wide range of pathological conditions. For instance, in immune thrombocytopenia, increased platelet apoptosis and autophagy have been observed, contributing to enhanced platelet destruction [19]. Similarly, in diabetes mellitus, platelet mitochondrial dysfunction represents a key mechanism linking hyperglycemia to increased apoptotic susceptibility [18]. The involvement of platelet apoptosis extends to cardiovascular pathologies, where it may serve as a link between hemostatic disorders and the progression of vascular damage [20,21]. Notably, certain biologicals and therapeutic agents have also been shown to modulate platelet apoptotic pathways, raising potential implications for treatment safety and efficacy in patients with systemic diseases [17]. Collectively, these findings underscore the clinical relevance of platelet apoptosis beyond its role in normal platelet turnover.

The relationship between these mechanisms and the development of coronary artery disease requires further study to identify significant markers that can predict the course of disease, assess postoperative risk, and prevent cardiovascular complications in patients who have undergone coronary artery bypass surgery.

The aim of this study was to evaluate the dynamics of platelet apoptosis before surgery and in the early postoperative period in patients undergoing coronary artery bypass grafting.

## 2. Results

Phosphatidylserine is a key component of the cell membrane that is located in the inner layer in healthy cell, but translocated to the outer layer of the membrane during apoptosis. This process is one of the early signs of cell death and can be used as a biomarker for apoptosis. To detect phosphatidylserine, a fluorescently labeled protein annexin V is used, which binds to it in the presence of calcium ions.

Another important parameter for studying apoptosis is the mitochondrial membrane potential which decreases during apoptosis and can be measured using the cationic dye JC-1. In a healthy cell with high mitochondrial potential, the dye accumulates in the mitochondria and forms aggregates that emit orange light. The low membrane potential prevents the accumulation of the dye, and it remains in the cytoplasm in its monomeric form, emitting a green glow. This shift in fluorescence from orange to green is a sensitive indicator of mitochondrial dysfunction and an early sign of apoptosis.

Along with the assessment of apoptosis, the platelet activation is also analyzed. It involves the rapid transport of adhesion molecules like p-selectin from intracellular granules to the cell surface in response to various stimuli. This process is detected using monoclonal antibodies.

### 2.1. Markers of Platelet Activation and Apoptosis Studied by Flow Cytometry

**Figure 1:**
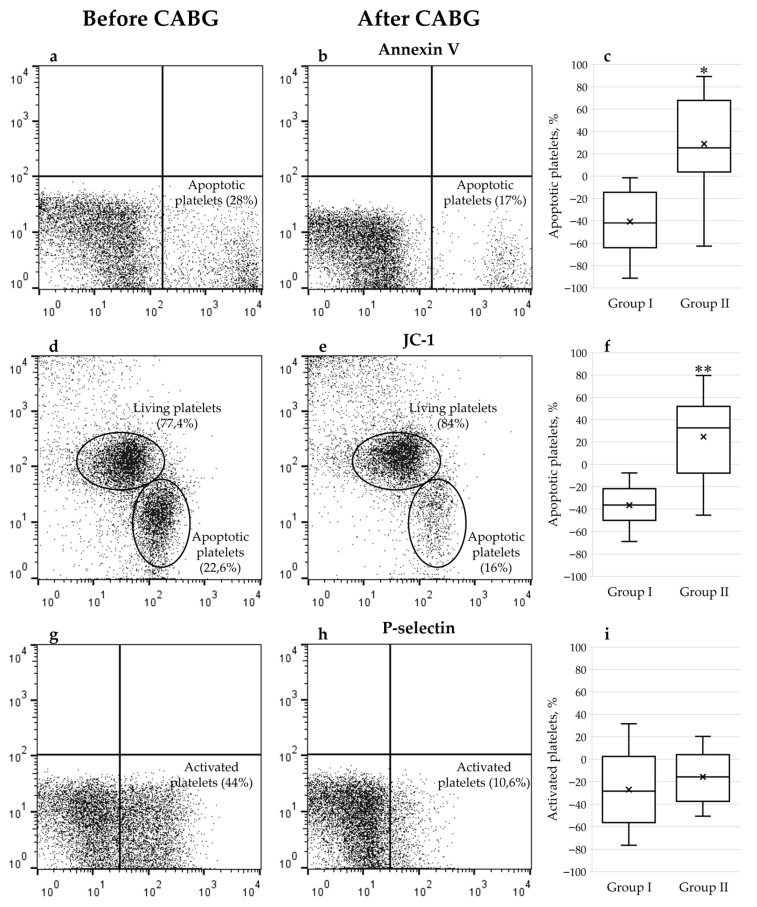
Representative histograms (**a**,**b**,**d**,**e**,**g**,**h**) and graphs (**c**,**f**,**i**) demonstrate the results. Box plots show the median (central line), the mean (cross), interquartile range (box), and whiskers extending to the minimum and maximum values within 1.5 × IQR. Number of apoptotic/activated platelets measured by flow cytometry was expressed as fold change compared to the state before CABG (*n* = 30). *—indicates a significant difference in number of apoptotic platelets before and after surgery measured using Annexin V between groups (adjusted *p* < 0.05); **—indicates a significant difference measured using JC-1 (adjusted *p* < 0.05).

Based on a comprehensive analysis of the functional state of platelets in patients after coronary bypass surgery, as described in Section 4, two groups were identified. Group I showed a significant decrease in the number of platelets undergoing apoptosis (59.35 ± 26.88% compared to the state before surgery vs. 121.98 ± 53.47%, adjusted *p* < 0.05), which was assessed using annexin V (Figure 1). The JC-1 dye in patients of the group I also showed a decrease in the number of platelets in the state of apoptosis compared to group II (63.46 ± 19.35% vs. 126.97 ± 46.26%, adjusted *p* < 0.05). Using antibodies to p-selectin, a decrease in the number of activated platelets was detected in patients after surgery in both groups, although there was no statistically significant difference between them (79.95 ± 42.72% vs. 84.34 ± 24.43%, *p* > 0.05). Thus, it can be noted that the number of platelets undergoing apoptosis varies and depends on the individual condition of each patient, while the number of activated platelets decreases after CABG independently of it.

### 2.2. Expression of Apoptosis-Related Proteins

**Figure 2:**
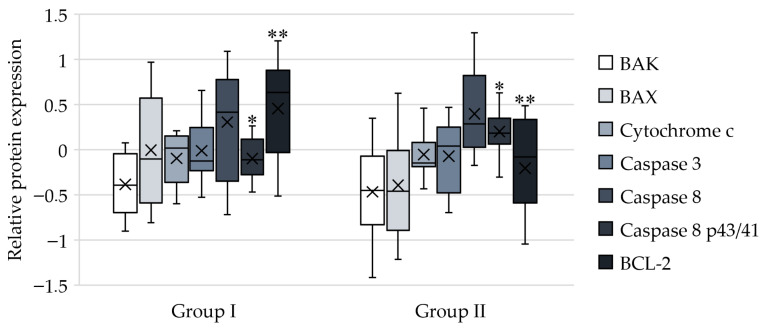
Graphs demonstrate the results of Western blot. Box plots show the median (central line), the mean (cross), IQR (box), and whiskers extending to the minimum and maximum values within 1.5 × IQR. Relative protein expression measured by Western blot is presented as log2-transformed fold change relative to the state before CABG (*n* = 30). *—indicates a significant difference in caspase 8 p43/41 expression before and after surgery between groups (adjusted *p* < 0.05); **—indicates a significant difference in BCL-2 expression (adjusted *p* < 0.05).

A number of proteins involved in apoptosis, such as BAX, BAK, caspase 3, caspase 8, cytochrome C, and BCL-2, were studied using Western blot analysis. The expression of caspase 8 cleavage products (p43/41) showed a statistically significant difference between two groups (0.902 ± 0.251 vs. 1.203 ± 0.193, adjusted *p* < 0.05), indicating the activation of pro-caspase 8 and the initiation of cell death through the extrinsic pathway of apoptosis. Additionally, there was a significant difference in the expression of BCL-2 (1.457 ± 0.492 vs. 0.805 ± 0.402, adjusted *p* < 0.05), with changes opposite to those in caspase 8 which also indicated activation of the extrinsic apoptotic pathway (Figure 2). The changes were consistent with flow cytometry data. A decrease in Caspase 8 expression and an increase in BCL-2 were observed in the group I, while reverse changes were seen in the group II.

### 2.3. mRNA Expression of the Apoptosis-Related Genes

**Figure 3:**
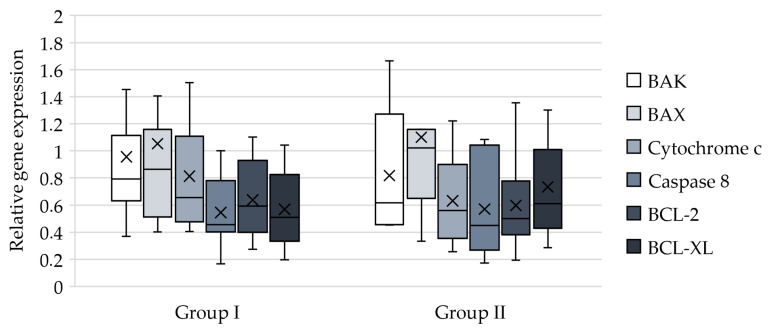
Graphs demonstrate the results of PCR. Box plots show the median (central line), the mean (cross), IQR (box), and whiskers extending to the minimum and maximum values within 1.5 × IQR. Relative gene expression measured by PCR is presented as fold change compared to the state before CABG (*n* = 30).

In addition, the expression of mRNA for the same proteins was evaluated. Although platelets do not synthesize mRNA de novo, they inherit it from their precursors, megakaryocytes. It is likely that changes occurring in the body after surgery affect the state of megakaryocytes, but these changes do not seem to be reflected in platelets, as no significant differences were found between the two groups. However, attention should be drawn to the decrease in the expression of mRNA responsible for both apoptosis and its inhibition, which was observed in both groups (Figure 3).

Thus, a group of patients with platelet apoptosis indicators remaining unchanged or even worsening was identified. Changes in platelet membrane and depolarization of mitochondrial membrane were observed, as well as expression of active caspase 8, indicating the activation of apoptosis through an extrinsic receptor-dependent signaling pathway.

Despite mitochondrial depolarization, the intrinsic apoptosis pathway is unlikely to be activated, as there have been no changes in protein expression responsible for apoptosis along this pathway. Interestingly, the level of platelet activation decreased in both groups, correlating with standard clinical examinations performed in all patients during treatment. Based on the above results, it can be assumed that platelet apoptosis is related to the course of coronary heart disease. Additional research and comparison with clinical data in the long term are required to assess the possibility of using this phenomenon in clinical practice.

## 3. Discussion

It is widely known that the state of platelets affects the course of coronary heart disease. Therapy aimed at suppressing platelet activity is an essential component of the treatment for these patients, regardless of whether surgical intervention is needed [3]. More aggressive therapy, involving antithrombotic medications that provide platelet quiescence, shows better results [22].

Research is ongoing to understand how the coronary artery disease itself affects platelet function and, consequently, how the disease progression can be predicted based on platelet status [20]. Surface markers on platelets, such as GPIIb/IIIa [23] and p-selectin [24,25], are being investigated because their presence is directly linked to platelet activation and clot formation. These data correlate with our findings: after treatment, the number of resting platelets increases, but it is not clear whether this can be used to predict outcome. Studies of circulating molecules associated with platelets, such as matrix metalloproteinases [26,27,28], and SCUBE1 [29], are being conducted. Even routinely measured parameters such as platelet size, volume variability [30], and relative abundance among blood cells are carefully analyzed for their prognostic significance.

Inflammatory biomarkers, such as platelets-to-lymphocytes ratio [31] and platelets-to-hemoglobin ratio [32], look particularly promising. Research is mainly conducted on routinely measured indicators in patients with various diseases, including those of the cardiovascular system. Although these biomarkers may be convenient for use in clinical practice, they may have low specificity, as many factors can affect platelet number and size. Concomitant diseases such as diabetes mellitus [33] and acute kidney injury [34] can also affect the accuracy of these biomarkers. More specific platelet properties, such as apoptosis or activation, seem more promising because they reflect the current state of the disease and are directly involved in its pathogenesis. Studies of the general proteome [35] of platelets are also underway to detect proteins specific to coronary heart disease and myocardial infarction. A number of genes have been identified whose expression is also increased in these diseases [36,37]. More recently, Talin-1/aIIbb3-mediated signaling pathway has been identified that seems to play a crucial role in the pathogenesis of coronary artery disease [38].

One of the methods of predicting adverse events is the systemic inflammation response index (SIRI) based on neutrophil, lymphocyte, and monocyte counts in peripheral blood [39]. However, it has recently been shown that including platelets in this index, which was named systemic immune-inflammation response index (SIIRI), increases its sensitivity for patients with coronary artery disease [40,41], especially those who have undergone CABG [42]. Changes in the functional state of platelets have been observed in patients with CAD compared to healthy volunteers [43], but there is little data on the relationship between platelets and treatment of CAD. The results obtained complement and extend the data presented previously. Expression of caspase 8 (p43/41) and phosphatidylserine translocation appear to be the most promising indicators of apoptosis, particularly in relation to platelets [43,44]. For the first time, the apoptosis of platelets in patients who underwent CABG was comprehensively studied using flow cytometry and Western blot, and individual differences among these patients were identified. The most significant limitation of this study is the absence of long-term follow-up for the current group of patients. Another limitation is the small number of participants, all of whom are men, both of which will be addressed in future research. The preoperative treatment, as well as the use of heparin and protamine during surgery, may have influenced the platelet condition, but since all patients received the same treatment in accordance with clinical guidelines we believe this impact did not significantly affect the results.

Spontaneous activation of platelets during isolation and processing, as well as the possibility of contamination by leukocytes, present significant challenges for their study. While we have taken measures to address these issues, such as using gentle isolation protocols and implementing light microscopy to control contamination, we cannot completely exclude some degree of preparation-induced artifact.

The data obtained suggest significant changes in platelet metabolic processes, which could play a crucial role in the development of CAD. These findings emphasize the need for a more comprehensive approach to assessing platelet function that goes beyond traditional methods.

Of particular importance is the investigation of platelet energy metabolism and its connection to thrombosis and inflammation in coronary heart disease. Future research in this field will allow for the implementation of standardized diagnostic protocols and personalized treatment strategies for patients with coronary pathologies in clinical practice.

## 4. Materials and Methods

### 4.1. Patient Characteristics

This study was conducted in accordance with the ethical principles of the Declaration of Helsinki of the World Medical Association (1964, 2004). Voluntary informed consent in writing from all patients was obtained. The study was approved by the ethics committee of the Moscow Regional Research Clinical Institute (No. 13427-c).

The study involved 30 patients suffering from coronary artery disease. The inclusion criteria were as follows according to the 2018 ESC/EACTS Guidelines on Myocardial Revascularization: the presence of hemodynamically significant stenosis of the main coronary arteries; a scheduled coronary artery bypass graft (CABG); age of 45–65 years; and the obtaining of informed consent. Acute myocardial infarction; chronic heart failure of functional class III–IV; cancer; and blood diseases were the exclusion criteria. The majority of patients exhibited generalized atherosclerotic disease of the coronary arteries. All patients underwent an examination, which included an assessment of physical status, clinical and biochemical tests, electrocardiogram, and echocardiography. All diagnoses of coronary heart disease by means of coronary angiography were confirmed. Demographic information about the participants is provided in Table 1.

Blood samples were taken from the cubital veins of patients (~9 mL) using a 4.5 mL disposable syringe containing sodium citrate (3.8%). Samples were processed no later than one hour after venipuncture.

Blood sampling from patients was carried out on the day before the operation, and 3–5 days after surgery. All patients received treatment according to the standard guidelines. At the time of blood sampling, all patients were hemodynamically stable.

As shown in a previous study, the number of platelets undergoing apoptosis, as measured using Annexin V, decreased after surgery in most patients [43]. This decrease was approximately 20%, but there was a small group of patients in whom the rate of apoptosis did not decrease [45].

Based on the data obtained using flow cytometry and specifically the expression of phosphatidylserine, these patients were divided into two groups: those with a significant decrease in platelet apoptosis and those with maintained or even increased apoptosis. These were group I (*n* = 19) and group II (*n* = 11) respectively. These two groups were then compared using flow cytometry, Western blot, and PCR.

### 4.2. Washed Platelet Preparation

Platelet-rich plasma (PRP) was separated by centrifugation (200× *g*, 10 min). Platelets were pelleted from PRP by centrifugation (800× *g*, 20 min) and re-suspended in HEPES buffered saline (10 mM HEPES, 135 mM NaCl, 3 mM KCl, 0.34 mM NaH_2_PO_4_, 1 mM MgCl_2_, 2.6 mM H_2_O, (pH 7.4), supplemented with 0.9 mg/mL D-glucose) at 2 × 10^9^ platelets/mL. While counting the platelets using a light microscopy (×400) analysis in a Goryaev chamber, the leukocytes contamination was checked and no leukocytes were detected in the entire field of view, resulting in a leukocyte count of less than 111 cells/μL.

### 4.3. Analysis Expression of CD61 by Flow Cytometry

**Figure 4:**
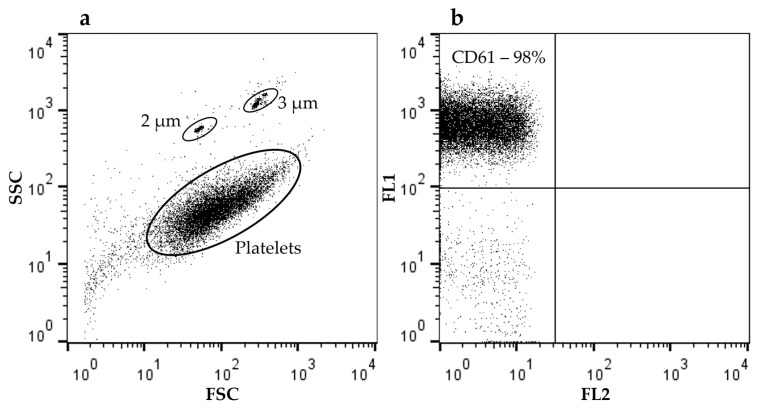
Expression of CD61 on the surface of the patients’ platelets. Sub-figure (**a**) shows cytometric analysis of platelet size distribution (FSC) and granularity (SSC) as well as calibration beads 2 and 3 µm; (**b**) shows staining of platelets with CD61/FITC antibodies.

The CD61 antigen (beta III integrin) is present in all normal, resting and activated platelets. The population of platelets was identified on the basis of particle size (forward and side scatter) compared with the MACSQuant Calibration Beads 2 and 3 µm in size (Miltenyi Biotec, Bergisch Gladbach, Germany) and the association with CD61 using anti-human antibodies (CD61/FITC, clone VI-PL2, BD Biosciences, San Jose, CA, USA). Isotype control FITC Mouse IgG1 (BD Biosciences, San Jose, CA, USA) was used. The obtained gated platelets expressed CD61 on the surface in almost 100% of cases (Figure 4).

### 4.4. Analysis of Phosphatidylserine Exposure by Flow Cytometry

Platelets in the amount of 1 × 10^7^ were incubated in an Annexin V-FITC in binding buffer (BD Biosciences, San Jose, CA, USA) at room temperature for 15 min. After this, 400 μL of buffer was added and the sample was immediately examined using flow cytometry. Platelets treated with A23187 at a concentration of 10 μmol/L (Sigma, St. Louis, MO, USA), a non-physiological platelet agonist which causes phosphatidylserine exposure were used as a positive control.

### 4.5. Analysis of Mitochondrial Membrane Potential by Flow Cytometry

To assess the mitochondrial membrane potential, JC-1 Mitochondrial Membrane Potential Assay Kit (cat. No: G1515-100T, Servicebio, Wuhan, China) was used in accordance with the manufacturer’s instructions. The required amount of JC-1 working solution was added to platelets in the amount of 1 × 10^7^, incubated in a CO_2_ incubator for 30 min, and immediately examined using flow cytometry. Platelets treated with carbonyl cyanide m-chlorophenyl hydrazone (CCCP) at a concentration of 100 μmol/L from the JC-1 kit was used as a positive control.

### 4.6. Analysis of Platelet Activation by Flow Cytometry

To assess the number of activated platelets, antibodies to p-selectin (PE Mouse Anti-Human CD62P, clone: AK-4, cat. No: 555524, BD Biosciences, San Jose, CA, USA) were used in accordance with the manufacturer’s instructions. Platelets treated with PE Mouse IgG1 (clone: MOPC-21, cat. No: 554680, BD Biosciences, San Jose, CA, USA) were used as an isotype control. Platelets treated with A23187 were used as a positive control. All the samples were analyzed using a flow cytometer FACSCalibur (Becton Dickinson, Franklin Lakes, NJ, USA). 50,000 events were accumulated for each sample. Data were collected using the CELLQuest program (Becton Dickinson, Franklin Lakes, NJ, USA).

### 4.7. Western Blotting

Platelets were lysed using Radio Immunoprecipitation Assay buffer and mixed with Laemmli sample buffer (4×) and heated to 100 °C. The purified proteins were analyzed using SDS-PAGE on 12% acrylamide gels using Mini-PROTEAN Tetra Cell (Bio-Rad Laboratories, Hercules, CA, USA) and electroblotted onto Bio-Rad 0.45 μM PVDF membrane (Bio-Rad Laboratories, Hercules, CA, USA). The membranes were then blocked with 5% milk (Blotting-grade blocker, Bio-Rad Laboratories, Hercules, CA, USA) in TBST buffer at RT for 1 h and then treated overnight at 4 °C with working dilutions of antibodies in the presence of 5% milk with 0.06% NaN_3_ in TBST buffer. The antibodies used were BAK (polyclonal, cat. No: FNab00796, FineTest, Wuhan, China), BAX (clone: 5F4, cat. No: FNab00811, FineTest, Wuhan, China), Caspase 3 (polyclonal, cat. No: FNab01289, FineTest, Wuhan, China), Caspase 8 (clone: 2C2, cat. No: FNab01294, FineTest, Wuhan, China), Cytochrome c (clone: ARC1153, cat. No: A4912, Abclonal, Woburn, MA, USA), BCL-2 (polyclonal, cat. No: FNab00839, FineTest, Wuhan, China), GAPDH (polyclonal, cat. No: AC001, Abclonal, Woburn, MA, USA). After that, the membranes were washed with TBST, and then incubated with a secondary horseradish peroxidase-conjugated goat anti-mouse IgG antibody (cat. No: FNSA-0003, FineTest, Wuhan, China) and anti-rabbit IgG antibody (cat. No: FNSA-0004, FineTest, Wuhan, China) for 1 h at RT.

The detection of bands was performed using an Odyssey XF Imaging System LI-COR Biosciences image station (LI-COR Biosciences, Lincoln, NE, USA) and a Hypersensitive chemiluminiscence ECL kit (FineTest, Wuhan, China), following the manufacturer’s instructions.

### 4.8. mRNA Isolation and Real-Time PCR Amplification

Total RNA was isolated using acid guanidinium thiocyanate–phenol–chloroform extraction method [46] with the ExtractRNA reagent (Evrogen, Moscow, Russia) following the manufacturer’s instructions. The concentration and purity of the nucleic acids were assessed using a Nanodrop 2000 spectrophotometer (Thermo Fisher, Waltham, MA, USA). DNase (Evrogen, Moscow, Russia) was used to process the samples, and cDNA synthesis was performed using MMLV RT kit (Evrogen, Moscow, Russia) according to the manufacturer’s instructions. Amplification was performed with primers (Table 2) and qPCR mix-HS SYBR (Evrogen, Moscow, Russia) using a Bio-Rad CFX-96 instrument (Bio-Rad, Hercules, CA, USA). Predesigned primer sequences for the target genes were obtained from OriGene Technologies (Rockville, MD, USA). The oligonucleotides were synthesized by Evrogen (Moscow, Russia). Prior to use, the primers were validated in silico for the absence of hairpin structures, primer–dimer formation as well as temperature (Tm) and GC content using OligoAnalyzer web tool (Integrated DNA Technologies; https://www.idtdna.com/pages/tools/oligoanalyzer; accessed on 15 January 2025). Specificity of each primer pair was confirmed by BLAST alignment against the human genome database (NCBI; https://blast.ncbi.nlm.nih.gov/Blast.cgi; accessed on 15 January 2025) to exclude off-target amplification. Results were analyzed using Bio-Rad CFX Manager (version 3.1; Bio-Rad Laboratories, Hercules, CA, USA) and Microsoft Excel (Microsoft Office 19, Redmond, WA, USA), applying the 2^−ΔΔCt^ method [47].

### 4.9. Statistics

The sample was normally distributed and evaluated using the Shapiro–Wilk test. Differences between groups were assessed using one-way analysis of variance. For each methodological approach, the Holm–Bonferroni correction was used to control for the family-wise error rate separately for each assay: flow cytometry (3 endpoints), Western blot (7 endpoints), and quantitative PCR (6 endpoints). A corrected *p*-value < 0.05 was considered statistically significant. The analysis was performed using StatSoft Statistica 12 for Windows.

## Figures and Tables

**Table 1:** Demographic and clinical characteristics of patients.

Characteristics	Patients (*n* = 30)
Age, years	58.9 ± 6.8
Men gender (*n*, %)	30 (100%)
BMI (kg/m^2^)	26 ± 3.1
Diagnosis	
two-vessel disease;	2 (7%)
three-vessel disease;	28 (93%)
Stenosis > 65%	30 (100%)
Left ventricular ejection fraction	
41–49%	3 (10%)
≥50%	27 (90%)
Operation	
Off-pump	28 (93%)
Internal mammary artery bypass	30 (100%)
Heart failure	4 (13%)
Hypertension	14 (47%)
Atrial fibrillation	3 (10%)
Diabetes mellitus	4 (13%)
Family history of CVD	18 (60%)
Smoking	21 (70%)

**Table 2:** Sequences of the primers used in PCR.

Gene	Forward Primer	Reverse Primer
*GAPDH*	GGAGCGAGATCCCTCCAAAAT	GGCTGTTGTCATACTTCTCATGG
*BAX*	CCCGAGAGGTCTTTTTCCGAG	CCAGCCCATGATGGTTCTGAT
*BAK*	ATGGTCACCTTACCTCTGCAA	TCATAGCGTCGGTTGATGTCG
*Cytochrome c*	AAGGGAGGCAAGCACAAGACTG	CTCCATCAGTGTATCCTCTCCC
*Caspase 8*	AGAAGAGGGTCATCCTGGGAGA	TCAGGACTTCCTTCAAGGCTGC
*BCL-2*	ATCGCCCTGTGGATGACTGAGT	GCCAGGAGAAATCAAACAGAGGC
*BCL-XL*	GCCACTTACCTGAATGACCACC	AACCAGCGGTTGAAGCGTTCCT

## Data Availability

Data is contained within the article or Supplementary Material.

## Supplementary Materials

The following supporting information can be downloaded at: https://www.mdpi.com/article/10.3390/ijms27073304/s1.

## Abbreviations

The following abbreviations are used in this manuscript:

CABG	Coronary artery bypass graft
CAD	Coronary artery disease
CCCP	Carbonyl cyanide m-chlorophenyl hydrazone
CVDs	Cardiovascular diseases
JC-1	(5,50,6,60-Tetrachloro-1,10,3,30-tetraethylbenzimidazolylcarbocyanine, iodide)
CD61	Cluster of differentiation 61
FITC	Fluorescein isothiocyanate
IQR	Interquartile range
mRNA	Messenger ribonucleic acid
ESC	European Society of Cardiology
EACTS	European Association for Cardio-Thoracic Surgery
PCR	Polymerase chain reaction
PRP	Platelet-rich plasma
SIRI	Systemic inflammation response index
SIIRI	Systemic immune-inflammation response index
