# Supplementary material for: Platelet Apoptosis in Patients with Coronary Artery Disease Before and After CABG
Source: Int J Mol Sci. 2026 Apr 6;27(7):3304. doi: 10.3390/ijms27073304 (PMC13072711; doi:10.3390/ijms27073304)
Supplement: Supplementary file 1 [file ijms-27-03304-s001.zip › ijms-4169330-supplementary.pdf]

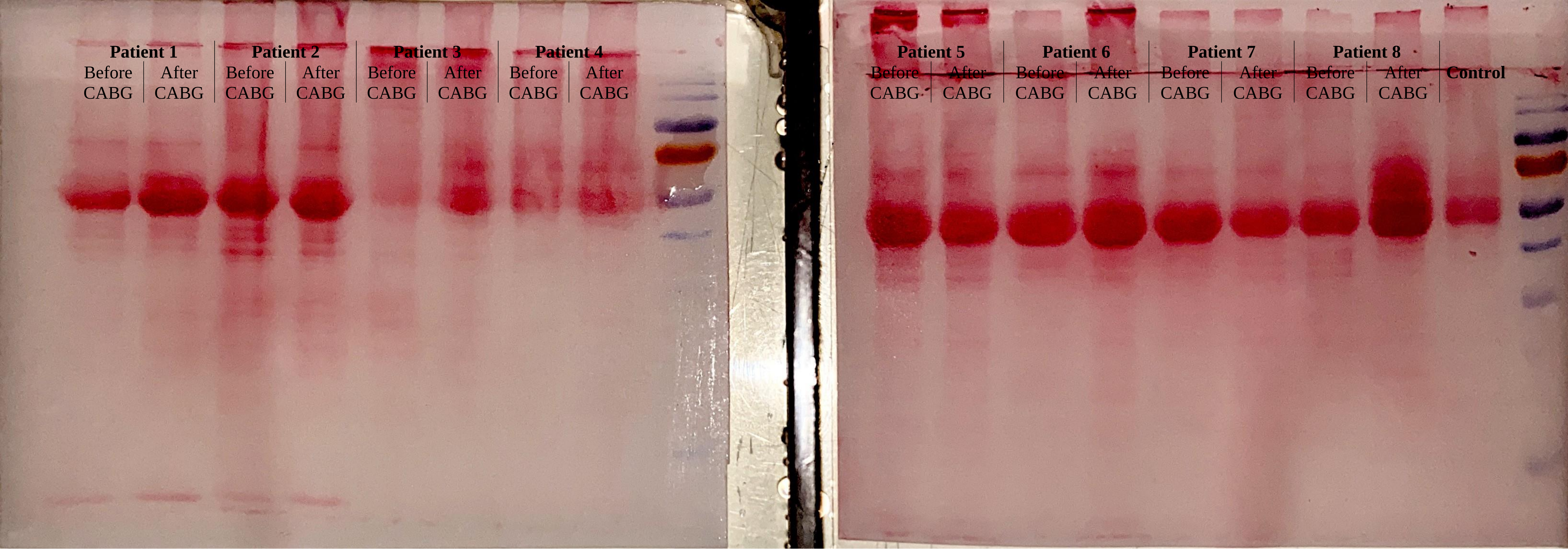

Patient 1
Before CABG
After CABG
Patient 2
Before CABG
After CABG
Patient 3
Before CABG
After CABG
Patient 4
Before CABG
After CABG
Patient 5
Before CABG
After CABG
Patient 6
Before CABG
After CABG
Patient 7
Before CABG
After CABG
Patient 8
Before CABG
After CABG
Control

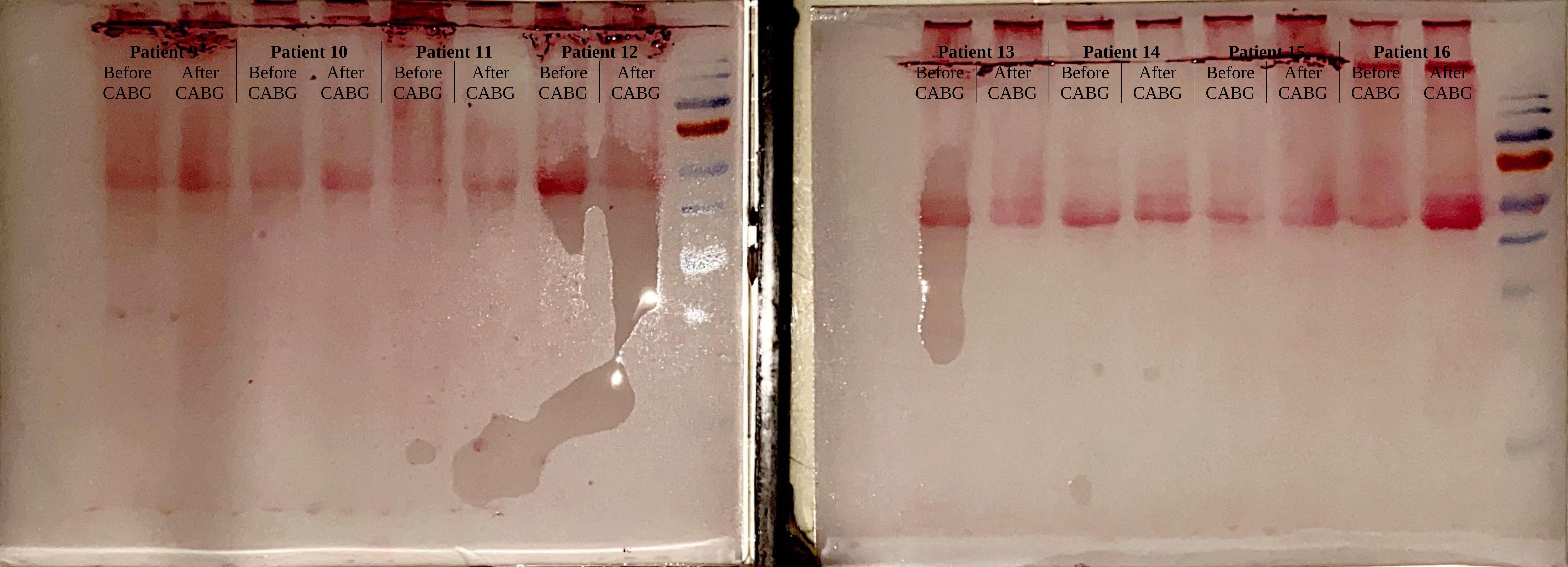

Patient 9
Before CABG
After CABG
Patient 10
Before CABG
After CABG
Patient 11
Before CABG
After CABG
Patient 12
Before CABG
After CABG
Patient 13
Before CABG
After CABG
Patient 14
Before CABG
After CABG
Patient 15
Before CABG
After CABG
Patient 16
Before CABG
After CABG

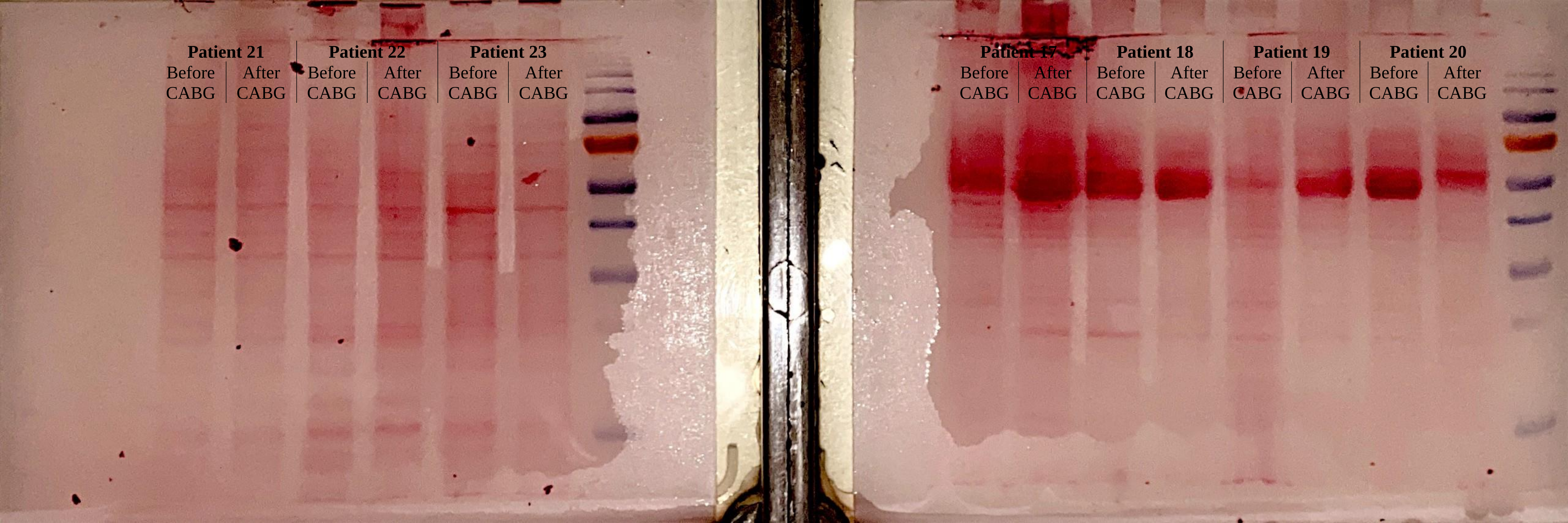
Patient 21
Before CABG
After CABG
Patient 22
Before CABG
After CABG
Patient 23
Before CABG
After CABG
Patient 17
Before CABG
After CABG
Patient 18
Before CABG
After CABG
Patient 19
Before CABG
After CABG
Patient 20
Before CABG
After CABG

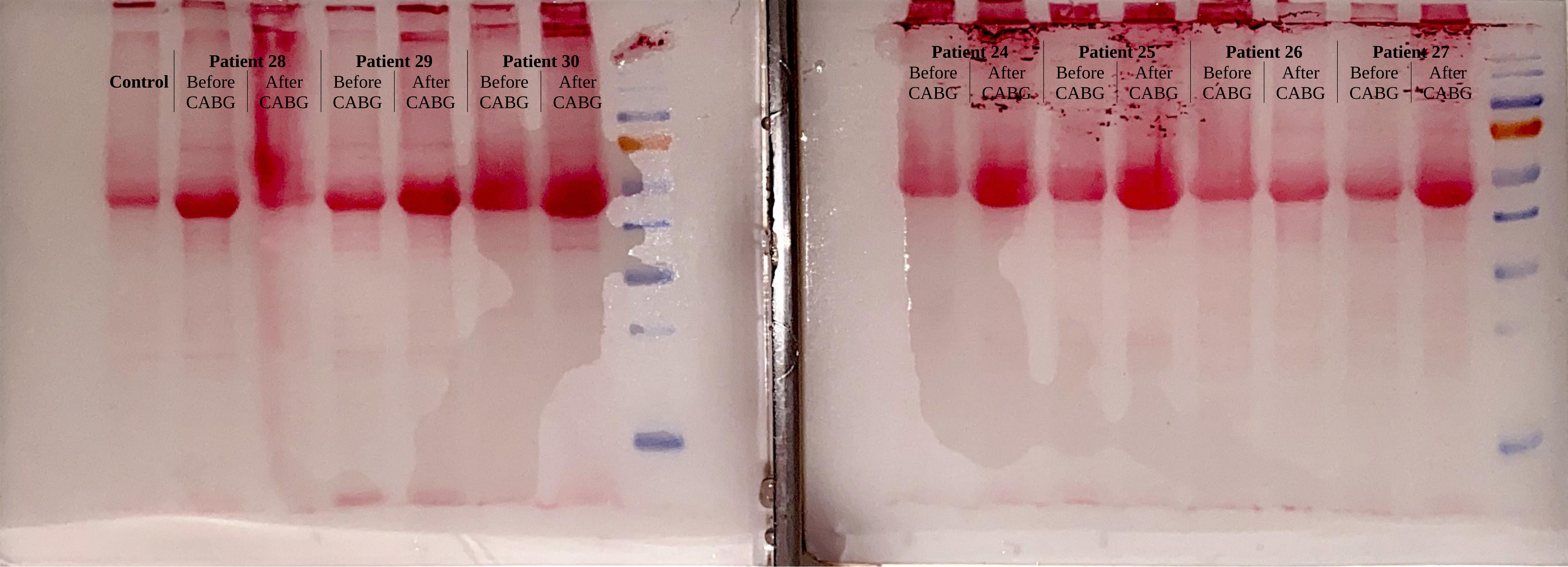
Control
Patient 28
Before CABG
After CABG
Patient 29
Before CABG
After CABG
Patient 30
Before CABG
After CABG
Patient 24
Before CABG
After CABG
Patient 25
Before CABG
After CABG
Patient 26
Before CABG
After CABG
Patient 27
Before CABG
After CABG
